# Associations Between Sleep Quality and Health Span: A Prospective Cohort Study Based on 328,850 UK Biobank Participants

**DOI:** 10.3389/fgene.2021.663449

**Published:** 2021-06-15

**Authors:** Muhammed Lamin Sambou, Xiaoyu Zhao, Tongtong Hong, Jingyi Fan, Til Bahadur Basnet, Meng Zhu, Cheng Wang, Dong Hang, Yue Jiang, Juncheng Dai

**Affiliations:** ^1^Department of Epidemiology, Center for Global Health, School of Public Health, Nanjing Medical University, Nanjing, China; ^2^Jiangsu Key Lab of Cancer Biomarkers, Prevention and Treatment, Collaborative Innovation Center for Cancer Personalized Medicine, Nanjing Medical University, Nanjing, China

**Keywords:** sleep quality, sleep score, health span, aging, population attributable risk percent, UK Biobank

## Abstract

**Objective:**

To examine the associations between sleep quality and health span using a prospective cohort design based on the UK Biobank (UKB).

**Materials and Methods:**

This longitudinal cohort study enrolled 328,850 participants aged between 37 and 73 years from UKB to examine the associations between sleep quality and risk of terminated health span. End of health span was defined by eight events strongly associated with longevity (cancer, death, congestive heart failure, myocardial infarction, chronic obstructive pulmonary disease, stroke, dementia, and diabetes), and a sleep score was generated according to five sleep behavioral factors (sleep duration, chronotype, sleeplessness, daytime sleepiness, and snoring) to characterize sleep quality. The hazard ratio (HR) and 95% confidence intervals (CIs) were calculated by multivariate-adjusted Cox proportional hazards model. Moreover, we calculated population attributable risk percentage (PAR%) to reflect the public health significance of healthy sleep quality.

**Results:**

Compared with poor sleep quality, participants with healthy sleep quality had a 15% (HR: 0.85, 95% CI: 0.81–0.88) reduced risk of terminated health span, and those of less-healthy sleep quality had a 12% (HR: 0.88, 95% CI: 0.85–0.92) reduced risk. Linear trend results indicated that the risk of terminated health span decreased by 4% for every additional sleep score. Nearly 15% health span termination events in this cohort would have been prevented if a healthy sleep behavior pattern was adhered to (PAR%: 15.30, 95% CI: 12.58–17.93).

**Conclusion:**

Healthy sleep quality was associated with a reduced risk of premature end of health span, suggesting healthy sleep behavior may extend health span. However, further studies are suggested for confirmation of causality and potential mechanism.

## Introduction

Health span is a significant phenotype that enables individuals to age in good health without chronic diseases or disability (Zenin et al., 2019). Although global life expectancy has increased (Gbd 2016 Mortality Collaborators, 2017), aging populations often suffer functional health loss, and absolute expansion of morbidity (Jagger et al., 2008; Gbd 2015 DALYs and Hale Collaborators, 2016). Due to the significance of sleep and the fact that humans spend one-third of their lives asleep, there is growing interest in sleep behavior as a determinant of health span (Kay and Dzierzewski, 2015). Moreover, an alarming number of individuals suffer from sleeping problems and sleep deprivation worldwide. It was estimated that over 36% of the global population are suffering from sleep loss (Kryger et al., 2017), and nearly 50–70 million Americans chronically suffer from sleeplessness and sleep-related disorders, which hinder daily functioning and adversely affect health and longevity (Institute of Medicine Committee on Sleep Medicine and Research, 2006).

Poor sleep moderates biological responses such as increased oxidative stress, altered inflammatory and coagulatory responses, neural autonomic control changes, and accelerated atherosclerosis (Kryger et al., 2017; Tobaldini et al., 2017), which also show the profound impact of sleep on maintaining individual health status. Recent studies revealed that sleep quality is associated with cardiometabolic health and mortality (Karthikeyan et al., 2019; Fan et al., 2020), as well as epigenetic and skin aging, frailty, and mental health (Lo et al., 2014; Oyetakin-White et al., 2015; Carskadon et al., 2019; Sun et al., 2020). Abnormal sleep duration (both short and long sleep duration) was associated with a higher risk of total cardiovascular diseases (CVDs), chronic heart disease (CHD), stroke, and myocardial infarction (MI) (Mesas et al., 2010; Cappuccio et al., 2011; Daghlas et al., 2019). Therefore, it is essential to pay particular attention to sleep problems.

Although the associations of sleep behavioral factors with morbidity and mortality risk are documented (Merikanto et al., 2013), the evidence related to health span is still insufficient and uncertain, especially from the perspective of integrating multiple sleep behaviors. Most of the studies were limited by their modest sample sizes, the inclusion of patients with certain diseases at baseline, short follow-up, or insufficient confounder control, leading to inconsistency in the findings (Cappuccio et al., 2011; Yin et al., 2017). To fill this void, we integrated eight predominant health span-terminating events (Zenin et al., 2019) and adopted a sleep score consisting of five sleep behavioral factors (chronotype, sleep duration, sleeplessness/insomnia, snoring, and daytime sleepiness) as a measurement for sleep quality. Therefore, our study aimed to assess the associations between sleep quality and health span based on a large-scale prospective cohort [UK Biobank (UKB)].

## Materials and Methods

### Study Population

The study population was composed of 328,850 participants of the UKB, a large-scale prospective cohort study with over 500,000 participants recruited between 2006 and 2010 across the United Kingdom. A detailed description of the UKB project is reported elsewhere (Sudlow et al., 2015). Briefly, the participants (aged from 37 to 73 years) attended one of 22 assessment centers in England, Wales, and Scotland, where they completed baseline questionnaires, underwent various physical assessments, and reported medical conditions. The North West Multicenter Research Ethical Committee approved the UKB project, and participants’ consent was obtained.

Before performing the analysis in this study, we pruned the data for suitability. First, we excluded 72,477 and 29,027 participants whose health span had terminated prior to the baseline according to in-patient hospital admissions data (UKB data category 2000) and self-reported diagnoses obtained *via* verbal interview (UKB data category 100074), respectively. Additionally, 72,153 participants with missing sleep-related data were excluded. Finally, 328,850 participants of the UKB were included in this study (Supplementary Figure 1).

### Ascertainment of Sleep Behaviors

The self-reported sleep behaviors (chronotype, sleep duration, sleeplessness/insomnia, snoring, and daytime sleepiness) were measured in the UKB using a standardized questionnaire. Except for sleep duration and snoring, the responses were measured on Likert scales from “never/rarely” to “usually” experiences (Fan et al., 2020). Chronotype means the tendency for earlier or later timing of sleep. An individual who prefers going to bed and waking earlier is considered a “morning person,” while a person who prefers going to bed and waking late is considered an “evening person” (Jones et al., 2019). Chronotype preference was assessed with the question “Do you consider yourself to be (i) “definitely a morning person,” (ii) “more a morning than evening person,” (iii) “more an evening than morning person,” or (iv) “definitely an evening person.” For sleep duration, participants responded to the question “About how many hours sleep do you get in every 24 h? (including naps)” with responses in hourly increments. Experience of sleeplessness/insomnia symptom was assessed with the question “Do you have trouble falling asleep at night or do you wake up in the middle of the night?” and the responses were given in 3-point Likert scale (never/rarely; sometimes; usually). Habitual snoring was assessed with the question “Does your partner or a close relative or friend complain about your snoring?” with responses of (i) yes or (ii) no. The question for subjective daytime sleepiness was “How likely are you to doze off or fall asleep during the daytime when you don’t mean to (for example, when working, reading, or driving)?,” with responses of (i) never/rarely, (ii) sometimes, (iii) often, or (iv) all of the time.

### Definition of Sleep Score and Sleep Quality

According to an epidemiologic study associated with sleep patterns and incident cardiovascular disease (Fan et al., 2020), for each sleep behavior, participants with the low-risk sleep behavior were assigned a score of 1, while those classified as high risk earn the score of zero (0). Then, all component scores were summed to acquire a total sleep score ranging from 0 to 5, with higher scores indicating healthier sleep patterns. Furthermore, we defined “sleep quality” as three levels: “healthy” (sleep scores 4–5), “less-healthy” (sleep scores 2–3), and “poor” (sleep scores 0–1) (Fan et al., 2020).

Here, the low-risk sleep behaviors include early chronotype (“morning” or “morning than evening”) (Merikanto et al., 2013), sleep duration 7–8 h per day (Gallicchio and Kalesan, 2009; Itani et al., 2017), never or rarely experience sleeplessness/insomnia symptoms (Hsu et al., 2015; Javaheri and Redline, 2017), no self-reported snoring (Li et al., 2014), and no frequent daytime sleepiness (“never/rarely” or “sometimes”) (Gangwisch et al., 2014).

### Ascertainment of Outcome

Health span is defined generally as aging without functional health loss (GBD, 2015; Li et al., 2020). In this study, health span was defined based on eight predominant health-terminating events strongly associated with longevity, such as congestive heart failure (CHF), myocardial infarction (MI), chronic obstructive pulmonary disease (COPD), stroke, dementia, diabetes, cancer, and death (Zenin et al., 2019). Health span was considered “terminated” for only participants first time diagnosed with any of these conditions during the UKB follow-up.

For each selected condition, except for cancer and death, we compiled a list of hospital data codes [International Classification of Diseases, 10th Revision (ICD-10)] and self-reported data codes (UKB data coding 6) to define these conditions in our study (Supplementary Table 1). We used the “National cancer registries linkage to UKB” (UKB data category 100092) to define cancer, and the “National death registries linkage to UKB” (UKB data category 100093) to define death event. The National cancer registries linkage to UKB was updated until December 14, 2016, earlier than the other two databases (inpatient hospital admissions data: March 31, 2017; National death registries linkage to UKB: February 14, 2018). To ensure consistency for the three databases, we set December 14, 2016, as the end date of follow-up in this study. Therefore, we calculated the personal follow-up time from the date of attending assessment center until the date of health span termination or December 14, 2016, whichever occurred first.

### Statistical Analysis

We applied descriptive statistics (mean, SD, and proportion) to explore the baseline characteristics of the participants and estimated multivariate-adjusted hazard ratio (HR) for terminated health span using Cox proportional hazards regression models (Chandola et al., 2010; Boden-Albala et al., 2012). The proportional hazards assumptions for the Cox model were tested using Schoenfeld residuals method (Weisberg, 2010). In the basic model, we adjusted for age, sex, and ethnicity and further adjusted, in the fully adjusted model, for Townsend Deprivation Index, education, body mass index (BMI), smoking status, alcohol consumption, physical activity, healthy diet, family history of diseases [cancer and cardiac–cerebrovascular disease (CCVD)], and medication (sleep-related drugs and aspirin/ibuprofen). More details of the covariates can be found in the section “Supplementary Method”). Furthermore, we calculated the population attributable risk percentage (PAR%) for high-risk sleep behaviors using the “epi2by2” function in “epiR” package of R (Stevenson et al., 2020). Stratified analyses were conducted according to age (<50, 50–60, and >60 years), gender (male and female), BMI (<30 and ≥30 kg/m^2^), smoking status (never and ever), alcohol intake frequency (≥once a week and <once a week), physical activity (low and moderate&high), healthy diet intake (yes and no), college degree (yes and no), and Townsend Deprivation Index (≥median and <median) to examine heterogeneity across these subgroups.

Additionally, in sensitivity analysis, we constructed a weighted sleep score of five sleep behaviors using the following equation: weighted sleep score = (β1 × factor 1 + β2 × factor 2 +. + β5 × factor 5) × (5/sum of the β coefficients) to evaluate the reliability of the results (Fan et al., 2020). To validate the robustness of our findings, we further performed sensitivity analyses: (1) excluding participants with terminated health span within the first 2 years of follow-up, (2) excluding those with poor self-reported health status at baseline, (3) further adjustment for principal components (PC1–3) and genotype chip. All analyses were performed using R (version 4.1.0), and statistical significance was defined as two-sided *p*-value ≤ 0.05.

## Results

In total, 49,772 participants of the 328,850 participants had terminated health span during the follow-up period, and approximately half of the events were caused by cancer (46.38%), followed by MI (17.73%) and death (10.99%) (Supplementary Table 2). The median follow-up time was 7.66 years (interquartile range: 6.80–8.42 years).

The baseline characteristics of 328,850 participants are summarized in Table 1. Overall, 4.08% of the participants had poor sleep quality (sleep scores 0–1), 57.59% had less-healthy sleep quality (sleep scores 2–3), and 38.33% had healthy sleep quality (sleep scores 4–5), with corresponding 18.87%, 15.75%, and 13.81% terminated health span, respectively. The female population was slightly higher among the healthy sleep quality group (58.45%). More participants with healthy (37.70%) sleep quality attained higher education than those with less-healthy (32.27%) or poor (27.89%) sleep quality. Besides, participants in the healthy sleep quality group had a relatively lower mean BMI (26.38 kg/m^2^), and approximately 37.64% of them engaged in high physical exercise. Participants who reported “currently smoking” seldom had healthy sleep quality (7.46%) compared to “never smoked” (61.45%). Participants with healthy sleep quality were more likely to have a healthy diet intake (79.15%) and less likely to have a family history of cardiovascular diseases (61.23%) and cancer (33.58%). Similarly, compared to poor sleep quality, participants with less-healthy and healthy sleep quality were less likely to take sleep-related drugs and aspirin/ibuprofen.

**TABLE 1:** Baseline characteristics of 328,850 participants according to sleep quality.

Characteristics (%)	Sleep quality		
	Poor (*n* = 13,432)	Less-healthy (*n* = 189,371)	Healthy (*n* = 126,047)
Terminated health span	2,535 (18.87)	29,834 (15.75)	17,403 (13.81)
Age, years, mean (SD)	55.32 (7.89)	55.91 (7.99)	55.35 (8.27)
Townsend Index, mean (SD)	−0.91 (3.26)	−1.41 (3.03)	−1.64 (2.89)
BMI, kg/m^2^, mean (SD)	29.22 (5.25)	27.50 (4.65)	26.38 (4.23)
Sex, female	6,359 (47.34)	1103,036 (54.41)	73,680 (58.45)
Ethnicity, white race	112,381 (92.18)	1179,379 (94.72)	1119,806 (95.05)
College or university degree	3,746 (27.89)	61,111 (32.27)	47,514 (37.70)
**Smoking status**			
Current smokers	2,320 (17.27)	21,568 (11.39)	9,397 (7.46)
Never smokers	6,077 (45.24)	1101,789 (53.75)	77,450 (61.45)
**Alcohol intake frequency**			
>3 times/week	5,915 (44.04)	87,335 (46.12)	55,260 (43.84)
Special occasions only/Never	2,665 (19.84)	32,431 (17.13)	22,404 (17.77)
**Physical activity**			
Low	2,841 (21.15)	30,115 (15.90)	15,907 (12.62)
High	3,754 (27.95)	61,972 (32.73)	47,442 (37.64)
Healthy diet	8,825 (65.70)	1140,048 (73.95)	99,760 (79.15)
Family history of CCVD	8,615 (64.14)	1119,497 (63.10)	77,184 (61.23)
Family history of cancer	4,909 (36.55)	66,667 (35.20)	42,323 (33.58)
Sleep-related drugs use	227 (1.69)	1,565 (0.83)	404 (0.32)
Aspirin/ibuprofen use	3,826 (28.48)	46,494 (24.55)	26,286 (20.85)
**Having low-risk sleep factors (%)**			
Early chronotype	879 (6.54)	93,740 (49.50)	1111,806 (88.70)
Sleep 7–8 h/day	548 (4.08)	1107,747 (56.90)	1119,576 (94.87)
Never/rarely insomnia	143 (1.06)	22,324 (11.79)	60,545 (48.03)
No self-reported snoring	612 (4.56)	96,133 (50.76)	1111,032 (88.09)
No frequent daytime sleepiness	10,639 (79.21)	1184,742 (97.56)	1125,777 (99.79)

*The chi-square test for categorical variables and Kruskal–Wallis test for continuous variables were used to calculate the *p* values across the sleep quality, and all the variables had *p* value < 0.001. SD, standard deviation; BMI, body mass index; CCVD, cardiac–cerebrovascular disease.*

In Table 2, associations for sleep quality with risk of terminated health span were exhibited. Compared to poor sleep quality, participants with healthy sleep quality and less-healthy sleep quality had 15% (HR: 0.85, 95% CI: 0.81–0.88) and 12% (HR: 0.88, 95% CI: 0.85–0.92) reduced risk of terminated health span, respectively. The corresponding PAR% for less-healthy and healthy sleep quality was 1.29% (PAR%: 1.29, 95% CI: 1.01–1.58) and 3.41% (PAR%: 3.41; 95% CI: 2.95–3.88), respectively.

**TABLE 2:** Associations for sleep quality with risk of terminated health span among 328,850 participants.

Sleep quality	Total N (%)	Cases N (%)	Basic model*	Fully adjusted model^†^	PAR% (95% CI)
				
			HR (95% CI)^a^	HR (95% CI)^a^	
Poor	13,432 (4.08)	2,535 (18.87)	ref	ref	ref
Less-healthy	189,371 (57.59)	29,834 (15.75)	0.80 (0.77–0.83)	0.88 (0.85–0.92)	1.29 (1.01–1.58)
Healthy	126,047 (38.33)	17,403 (13.81)	0.72 (0.69–0.75)	0.85 (0.81–0.88)	3.41 (2.95–3.88)

N, number; HR, hazard ratio; 95% CI, 95% confidence interval; ref, reference; PAR%, population attributable risk percentage; BMI, body mass index; CCVD, cardiac–cerebrovascular disease. *Basic model: adjusted for age, sex, and ethnicity. ^†^Fully adjusted model: additionally adjusted for Townsend Deprivation Index, education, BMI, smoking status, alcohol consumption, physical activity, healthy diet, family history of diseases (cancer and CCVD), and medication (sleep-related drugs and aspirin/ibuprofen). ^*a*^Each group was compared to participants with poor sleep quality.

**FIGURE 1:**
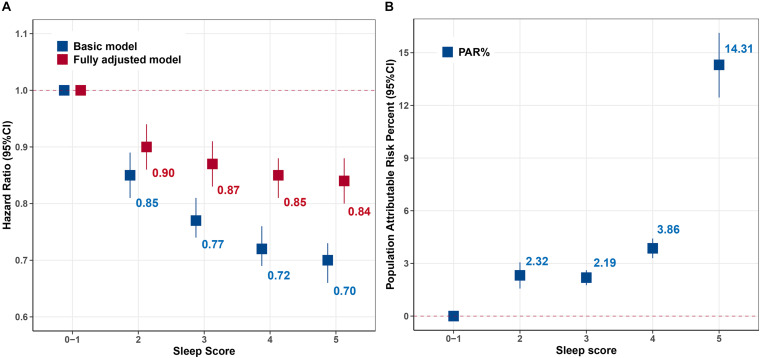
Hazard ratio (HR) and population attributable risk percentage (PAR%) for risk of terminated health span according to sleep score. **(A)** HR [95% confidence interval (95% CI)]. **(B)** Population attributable risk percentage (PAR%). Basic model: adjusted for age, sex, and ethnicity. Fully adjusted model further adjusted for Townsend Deprivation Index, education, body mass index (BMI), smoking status, alcohol consumption, physical activity, healthy diet, family history of diseases [cancer and cardiac–cerebrovascular disease (CCVD)], and medication (sleep-related drugs and aspirin/ibuprofen). Sleep scores 0–1 were the reference. The values were the point estimation of HRs and PAR%.

From the perspective of sleep score, we found the participants with the score 5 had the lowest risk of premature end of health span (HR: 0.84, 95% CI: 0.80–0.88), and the trend analysis also revealed that the risk of terminated health span decreased by 4% (HR: 0.96, 95% CI: 0.96–0.97) for every additional sleep score (Figure 1A). Moreover, the corresponding PAR% for score 5 was nearly 15% (PAR%:14.31; 95% CI: 12.45–16.13) compared to those with the lowest sleep scores (Figure 1B).

**FIGURE 2:**
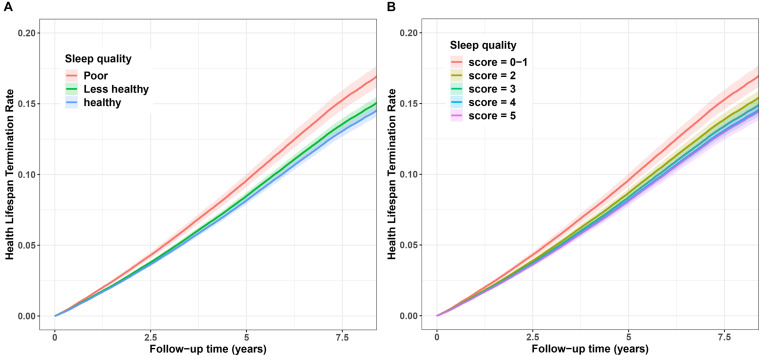
Cumulative hazard curve for the associations between sleep quality and risk of terminated health span. **(A)** Sleep quality (poor, less–healthy, and healthy). **(B)** Sleep score (scores 0–1, 2, 3, 4, and 5). The *Y*-axis represents cumulative hazard of terminated health span, the while *X*-axis represents the follow-up time (years). Shaded regions represent the 95% confidence intervals (95% CIs). Cumulative hazard curves were based on the fully adjusted model.

Additionally, we also demonstrated the cumulative hazard curves between sleep situation and terminated health span. Figure 2A showed that with increasing follow-up time, the cumulative hazard of terminated health span increased more in individuals with poor sleep quality than those with less-healthy or healthy sleep quality. Similar results were observed for sleep score, showing distinct risk between sleep scores 0–1 and high scores (Figure 2B).

Then, we further explored the effects of related sleep traits on health span (Table 3). Participants with the low-risk sleep behaviors such as “sleep duration 7–8 h/day” (HR: 0.94, 95% CI: 0.92-0.95), “Never/rarely insomnia” (HR: 0.94, 95% CI: 0.92–0.96), and “rarely daytime sleepiness” (HR: 0.83, 95% CI: 0.79–0.87) had decreased risk of terminated health span. Furthermore, PAR% for terminated health span suggests that nearly 15% (PAR%: 15.30, 95% CI: 12.58–17.93) of terminated health span in this cohort would not have occurred if all participants had been in the low-risk group for all five sleep factors.

**TABLE 3:** Associations for low-risk sleep behaviors with risk of terminated health span among 328,850 participants.

Sleep behaviors	Total (N)	Cases (N)	Basic model*	Fully adjusted model†	PAR% (95% CI)
				
			HR (95% CI)^a^	HR (95% CI)^a^	
Early chronotype	206,425 (62.77)	31,686 (15.35)	0.95 (0.94–0.97)	1.00 (0.98–1.01)	−1.42 (−2.04 to −0.80)
Sleep 7–8 h/day	227,871 (69.29)	33,049 (14.50)	0.88 (0.87–0.90)	0.94 (0.92–0.95)	4.17 (3.62–4.72)
Never/rarely insomnia	83,012 (25.24)	11,510 (13.87)	0.92 (0.90–0.94)	0.94 (0.92–0.96)	8.39 (7.02–9.74)
No self-reported snoring	207,777 (63.18)	29,664 (14.28)	0.93 (0.91–0.95)	0.99 (0.97–1.01)	5.67 (5.04–6.30)
Rarely daytime sleepiness	321,158 (97.66)	48,208 (15.01)	0.77 (0.73–0.81)	0.83 (0.79–0.87)	0.82 (0.68–0.96)
All five factors (overall)^b^	24,548 (7.46)	3,147 (12.82)	0.89 (0.86–0.92)	0.96 (0.93–1.00)	15.30 (12.58–17.93)

*Basic model: adjusted for age, sex, and ethnicity. ^*a*^Compared with all other participants not in this low-risk group. ^*b*^All low-risk factors were included simultaneously in the same model, and participants without all five low-risk behaviors were set as the reference ^†^Fully adjusted model: additionally adjusted for Townsend Deprivation Index, education, BMI, smoking status, alcohol consumption, physical activity, healthy diet, family history of diseases (cancer and CCVD), and medication (sleep-related drugs and aspirin/ibuprofen).

In stratified analyses, we observed that the associations of sleep quality with the risk of terminated health span were largely consistent across subgroups, except the smoking status. The ever smokers had a stronger association than never smokers (Supplementary Figure 2). Additionally, we constructed a weighted sleep score to reevaluate its association with the risk of terminated health span. We found that high-grade weighted sleep score (weighted sleep score 4∼5 vs. 0∼<1, HR: 0.76, 95% CI: 0.71–0.82) also reduced the risk of terminated health span (Table 4).

**TABLE 4:** Associations for weighted sleep score with risk of terminated health span among 328,850 participants.

Weighted score	Total N (%)	Cases N (%)	Basic model*	Fully adjusted model^†^
			
			HR (95% CI)^a^	HR (95% CI)^a^
0∼ < 1	3,409 (1.04)	746 (21.88)	ref	ref
1∼ < 2	3,864 (1.18)	749 (19.38)	0.83 (0.75–0.92)	0.89 (0.81–0.99)
2∼ < 3	28,229 (8.58)	5,055 (17.91)	0.80 (0.74–0.86)	0.84 (0.77–0.90)
3∼ < 4	120,549 (36.66)	19,418 (16.11)	0.72 (0.67–0.78)	0.79 (0.74–0.85)
4∼5	172,799 (52.55)	23,804 (13.78)	0.65 (0.60–0.70)	0.76 (0.71–0.82)
Overall (continuous)	328,850 (100.00)	49,772 (15.14)	0.91 (0.90–0.92)	0.94 (0.93–0.95)

N, number; HR, hazard ratio; 95% CI, 95% confidence interval; ref, reference; BMI, body mass index; CCVD, cardiac–cerebrovascular disease. *Basic model: adjusted for age, sex, and ethnicity. †Fully adjusted model: additionally adjusted for Townsend Deprivation Index, education, BMI, smoking status, alcohol consumption, physical activity, healthy diet, family history of diseases (cancer and CCVD), and medication (sleep-related drugs and aspirin/ibuprofen). ^*a*^Each group was compared to participants with 0–1 sleep scores.

Moreover, further sensitivity analyses were performed by respectively excluding participants with terminated health span within the first 2 years of follow-up and those with poor self-reported health status at baseline. The associations were largely similar to our previous findings (Supplementary Tables 4, 5). Given that complicated structure of population in UKB, we additionally adjusted the top 3 principal components (PC1–3) and genotype chip to offset the potential effect. Similarly, the results were consistent and supported the robustness of the observed findings in our study (Supplementary Table 6).

## Discussion

In this large-scale prospective cohort study, we examined the associations of sleep quality/sleep score with risk of terminated health span based on 328,850 participants of the UKB. Participants with a healthy sleep quality had a 15% lower risk of terminated health span. The PAR% further suggested that nearly 15% of terminated health span in this cohort would not have occurred if all participants had low-risk sleep behavior for all five sleep behavioral factors. Besides, four sensitivity analyses implemented in this study indicated that the associations we found are robust and reliable to some degree.

Our results are in line with other comparable findings that sleep behavior affects health and wellbeing (Cappuccio et al., 2011; Gangwisch et al., 2014). Although sleep behavioral factors separately have a bearing on health, it was significant to evaluate a combination of sleep behavioral factors due to their synchrony that could jointly increase the risk of health span termination (Javaheri and Redline, 2017). For instance, a previous study showed that insomnia/sleeplessness was related to sleep duration and excessive daytime sleepiness (EDS), and late chronotype reduced sleep duration (Depner et al., 2014). Thus, we generated a sleep score integrating five sleep behaviors to comprehensively assess sleep quality and its association with health span, which was characterized by a host of eight health events that are commonly involved in health span termination (Zenin et al., 2019). Our study showed that healthy sleep quality reduced the risk of terminated health span, suggesting that healthy sleep behavior can improve health span. In agreement with our finding, previous studies showed that insomnia accompanied short sleep duration (Hsu et al., 2015; He et al., 2017; Javaheri and Redline, 2017), and habitual snoring with EDS increased the risk of hypertension, lung cancer (Liu et al., 2019; Campos et al., 2020), vascular death (Blachier et al., 2012; Boden-Albala et al., 2012), atherosclerosis (Sands et al., 2013; Javaheri and Redline, 2017), and diabetes (Li et al., 2015).

We also observed a decreased risk of terminated health span for single low-risk sleep behaviors, such as “sleep duration 7–8 h/day,” “no daytime sleepiness,” “never/rarely insomnia/sleeplessness,” and “early chronotype.” Similarly, high-risk sleep behavioral factors including “late chronotype” (Merikanto et al., 2013; Erren et al., 2016), “abnormal sleep duration” (Mesas et al., 2010; Cappuccio et al., 2011; Rudnicka et al., 2017; Daghlas et al., 2019), “frequently experience insomnia/sleeplessness” (Hsu et al., 2015; Javaheri and Redline, 2017), “habitual snoring” (Seidel et al., 2012; Sands et al., 2013; Li et al., 2015), and “excessive daytime sleepiness” (Blachier et al., 2012; Boden-Albala et al., 2012; Barfield et al., 2019) were associated with increased risk of chronic disease morbidity and mortality. If all these five high-risk sleep behaviors were rectified appropriately, nearly 15% of terminated health span would have been prevented, highlighting the importance of adhering to healthy sleep behaviors. However, it is worth noting that due to the multiple-center and large-scale design of UKB, these 328,850 participants aged from 37 to 73 years were nationwide. Besides, sleep traits were collected by trained volunteers according to a standard questionnaire. Therefore, the exposure distribution could represent the general population of United Kingdom, indicating the reliability of the PAR% we calculated at some degree, although a further validation in other cohorts was still necessary.

Biologically, sleep regulates many important pathways in the human physiology, such as autonomic, sympathetic, cardiometabolic, and immunologic responses (Cappuccio et al., 2011; Tobaldini et al., 2017), which support that adopting healthy sleep behavior to the circadian rhythm would enhance health and quality of life (Backhaus et al., 2015; Jones et al., 2019). On the other hand, poor sleep quality affects functional health in both young and adult, including disruption of cognitive performance and diurnal alertness (Skeldon et al., 2016). Moreover, it is essential to be cautious about shared and non-shared environmental determinants of ill-sleep habit (Gregory et al., 2016), including lifestyle, such as alcohol dependence, smoking, obesity, physical inactivity, and stress, that could upset healthy sleep behaviors (Chakravorty et al., 2016; Christie et al., 2016; Liao et al., 2019; Garcia-Marin et al., 2020).

Moreover, sleep disorders and poor sleep habits are alarming health threats warranting more public attention and are necessary to take appropriate action to promote sleep quality and health status, particularly for those with irregular sleep patterns, such as shift workers (Palermo et al., 2015; Redeker et al., 2019). A previous study among shift workers showed that resting and napping lowered the levels of sleepiness at the end of the shift (Barthe et al., 2015), which means more efficiency at work and less chances of accidents due to sleepiness (Ruggiero and Redeker, 2014; Geiger-Brown et al., 2016) and ultimately beneficial for the extension of health span. In addition to potential contributions to the individual’s life quality, a healthy sleep quality may also mitigate extravagant medical costs associated with chronic disease morbidity as well as lighten the heavy burden of social demands on health services. Therefore, we not only aim to investigate the potential effects of sleep quality on health span but also hope to call for more attention to individual sleep problems and correct improper sleep patterns as far as possible.

Here, the definition of health span we adopted is a promising longevity phenotype, reflecting individual aging and health status. Based on the richness and accuracy of clinical information in UKB, the construction of the health span phenotype is reliable and robust. Thus, we have a chance to assess the associations between sleep quality and risk of premature health span termination for the first time. The sleep score constructed by five sleep behaviors is an effective way to measure the sleep quality quantitatively. Meanwhile, the reliable data, large-scale sample, and long-term follow-up time of UKB provide sufficient power for our study. However, all the sleep behaviors are self-reported, which may lead to misclassification of exposures inevitably. To our knowledge, misclassification will underestimate the associations we observed. Although we have adjusted the sociodemographic characteristics, lifestyles, and other confounding factors in the full model, residual confounding from unknown or unmeasured factors still remains possible. Thus, the effects of associations and the PAR% we calculated are essential to be further validated in other perspective cohorts. Thirdly, a single measurement of sleep behaviors at baseline is not satisfactory to reflect the dynamic change of sleep factors during the following time, which means that the evaluation of effects of changing sleep patterns on health span requires repeated measurements of sleep traits. Moreover, in our observational study, the potential causality is hard to determine, and further work is necessary. Finally, most of the study participants are white, and generalizing the findings to other populations should warrant caution.

In summary, we tentatively explored the effect of sleep quality on health life span in this study. A healthy sleep quality plays an important role in individual health status, aging, and diseases. Sleep problem is not only related to individual physical and mental health but also a public health and social problem, which deserves more attention and early intervention.

## Conclusion

In this large-scale prospective study that enrolled 328,850 participants, we found that healthy sleep quality was associated with a reduced risk of premature end of health life span, suggesting that healthy sleep behaviors may be beneficial to extend health life span. Therefore, sleep problems deserve more attention and early intervention. However, further studies are suggested for confirmation of causality and potential mechanism.

## Data Availability Statement

The raw data supporting the conclusions of this article will be made available by the authors, without undue reservation.

## Ethics Statement

The studies involving human participants were reviewed and approved by the North West Multi-Centre Research Ethics Committee. The patients/participants provided their written informed consent to participate in this study.

## Author Contributions

JD contributed to the conception and design of the study. MS, XZ, and TH conducted the statistical analysis and wrote the first draft. TH, JF, and TB helped apply for the permission to use data and offered technical support during the study. MZ, CW, DH, and YJ critically revised the manuscript for important intellectual content. All authors reviewed and approved the final manuscript.

## Conflict of Interest

The authors declare that the research was conducted in the absence of any commercial or financial relationships that could be construed as a potential conflict of interest.
