# Supplementary material for: Associations Between Sleep Quality and Health Span: A Prospective Cohort Study Based on 328,850 UK Biobank Participants
Source: Front Genet. 2021 Jun 15;12:663449. doi: 10.3389/fgene.2021.663449 (PMC8239359; doi:10.3389/fgene.2021.663449)
Supplement: Supplementary file 1 [file Table_1.doc]

**Supplementary materials**

**Supplementary Method. Covariates assessment in the UKB**

We considered the following covariates for adjustment in our analyses: age (continuous), sex (male, female), ethnicity (white race, others), Townsend Deprivation Index (continuous), education (college or university degree, other degrees), BMI (continuous), smoking status (never, previous, current), alcohol consumption (never, special occasions only, one to three times a month, once or twice a week, three or four times a week, daily or almost daily), physical activity [International Physical Activity Questionnaire (IPAQ) activity group](high, moderate, low), healthy diet (yes, no), family history of cancer (yes, no), family history of CCVD (cardiac-cerebral vascular disease)(yes, no), sleep-related drugs use (yes, no) and aspirin/ibuprofen use (yes, no).

In addition, Townsend Deprivation Index is an area-based proxy measure for socioeconomic status provided in UKB (Fan et al., 2020). The International Physical Activity Questionnaire (IPAQ) is a standardized self-report questionnaire, aiming at estimating physical activity and sedentary behavior for adults aged 15-69 years (Cleland et al., 2018). Moreover, the definition of healthy diet patterns we adopted based on a study published on *British Medical Journal* (Rutten-Jacobs et al., 2018). Family history was generated by diseases records derived from parents and siblings (CCVD including stroke and heart diseases). Finally, the corresponding codes for sleep-related drugs (Daghlas et al., 2019) were listed in **Supplementary Table 3**.

In cases of missing information (including participants who marked “do not know,” or “prefer not to answer”), medians were imputed for continuous covariates, and a missing indicator was added to the model (Fan et al., 2020). All covariates had <1% missing, except physical activity (16.8%), family history of cancer (5.5%) and CCVD (4.2%).

**References**

Cleland, C., Ferguson, S., Ellis, G., and Hunter, R.F. (2018). Validity of the International Physical Activity Questionnaire (IPAQ) for assessing moderate-to-vigorous physical activity and sedentary behaviour of older adults in the United Kingdom. *BMC Med Res Methodol* 18(1)**,** 176. doi: 10.1186/s12874-018-0642-3.

Daghlas, I., Dashti, H.S., Lane, J., Aragam, K.G., Rutter, M.K., Saxena, R., et al. (2019). Sleep Duration and Myocardial Infarction. *Journal of the American College of Cardiology* 74(10)**,** 1304-1314. doi: 10.1016/j.jacc.2019.07.022.

Fan, M., Sun, D., Zhou, T., Heianza, Y., Lv, J., Li, L., et al. (2020). Sleep patterns, genetic susceptibility, and incident cardiovascular disease: a prospective study of 385 292 UK biobank participants. *Eur Heart J* 41(11)**,** 1182-1189. doi: 10.1093/eurheartj/ehz849.

Rutten-Jacobs, L.C., Larsson, S.C., Malik, R., Rannikmäe, K., Sudlow, C.L., Dichgans, M., et al. (2018). Genetic risk, incident stroke, and the benefits of adhering to a healthy lifestyle: cohort study of 306 473 UK Biobank participants. *Bmj* 363**,** k4168. doi: 10.1136/bmj.k4168.

**Abbreviation: CHF, congestive Heart Failure; COPD, chronic obstructive pulmonary disease; MI, myocardial Infarction.**

| **Supplementary Table 1. Disease codes for health span composition** | | |
| --- | --- | --- |
| **Diseases** | **ICD10 in-patient hospital  admissions data (UKB data category 2000)** | **Self-reported diagnoses obtained  via verbal interview (UKB data category 100074)** |
| CHF | I50 | 1076 |
| COPD | J44 | 1112 |
| MI | I21, I22, I23, I24, I25 | 1075 |
| dementia | F00, F01, F02, F03, F04, F05 | 1263, 1258, 1259, 1260, 1261, 1262 |
| diabetes | E10, E11, E12, E13, E14 | 1220, 1221, 1222, 1223, 1521 |
| stroke | I60, I61, I62, I63, I64 | 1081, 1086, 1491, 1583 |

| **Supplementary Table 2. Number of events derived from clinical data for selected diseases and combined number used for subsequent analysis for total 328,850 participants** | | | |
| --- | --- | --- | --- |
| **Outcomes** | **Participants  (n)** | **Percentage  (%)** | **Combined N**† **(%)** |
| Cancer | 29486 | 46.38 | 49772 (15.14) |
| MI | 11274 | 17.73 |
| Death | 6986 | 10.99 |
| Diabetes | 6074 | 9.55 |
| COPD | 4766 | 7.50 |
| Stroke | 2830 | 4.45 |
| Dementia | 1241 | 1.95 |
| CHF | 916 | 1.44 |
| Total N* | 63573 | 100.00 |  |

*: The total number represents the person times of all kinds of events occurred. Some participants may successively occur multiple outcome events.

†: The combined number is the actual number of people who had terminated health span.

Abbreviation: CHF, congestive Heart Failure; COPD, chronic obstructive pulmonary disease; MI, myocardial Infarction; N, number.

| **Supplementary Table 3. List of medications used in the study of Daghlas et al. to define the sleep medications covariate** | |
| --- | --- |
| **Sleep medication** | **Treatment/medication code (UKB data-field 20003)** |
| Oxazepam | 1140863442 |
| Meprobamate | 1140863378 |
| Medazepam | 1140863372 |
| Bromazepam | 1140863318 |
| Orazepam | 1140863302 |
| Clobazam | 1140863268 |
| Chlormezanone | 1140863262, 1140868274 |
| Temazepam | 1140863202 |
| Nitrazepam | 1140863182, 1140863104 |
| Lormetazepam | 1140863176 |
| Diazepam | 1140863152, 1141157496 |
| Zopiclone | 1140863144 |
| Triclofos sodium | 1140863140 |
| Methyprylone | 1140856040 |
| Prazepam | 1140855944 |
| Triazolam | 1140855914 |
| Ketazolam | 1140855860 |
| Dichloralphenazone | 1140855824 |
| Clomethiazole | 1140909798 |
| Zaleplon | 1141171404 |
| Butobarbital | 1141180444 |

| **Supplementary Table 4. Sensitivity analyses excluding participants with terminated health span within the first two years of follow-up (n=10,794) for the associations between sleep score and risk of health span termination.** | | | | |
| --- | --- | --- | --- | --- |
| **Sleep score** | **Total N (%)** | **Cases N (%)** | **Basic model^a^** | **Fully adjusted model^b^** |
|  |  |  | **HR (95%CI)** | **HR (95%CI)** |
| 1 | 12896 (4.05) | 1999 (15.5) | ref | ref |
| 2 | 61125 (19.22) | 8238 (13.48) | 0.84 (0.80-0.88) | 0.89 (0.85-0.94) |
| 3 | 121752 (38.28) | 15102 (12.40) | 0.77 (0.73-0.80) | 0.86 (0.82-0.90) |
| 4 | 98402 (30.94) | 11159 (11.34) | 0.72 (0.68-0.75) | 0.84 (0.80-0.88) |
| 5 | 23881 (7.51) | 2480 (10.38) | 0.69 (0.65-0.73) | 0.84 (0.79-0.89) |
| continuous | 318056 (100.00) | 38978 (12.26) | 0.92 (0.91-0.93) | 0.97 (0.95-0.98) |

N, number; Hazard ratio, HR; 95%CI, 95% confidence interval; ref, reference;

**^a^:** Basic model, adjusted for age, sex and ethnicity;

**^b^:** Fully adjusted model, additionally adjusted for Townsend Deprivation Index, education, BMI, smoking status, alcohol consumption, physical activity, healthy diet, family history of diseases (cancer and CCVD) and medication (sleep-related drugs and aspirin/ ibuprofen).

| **Supplementary Table 5. Sensitivity analyses excluding participants with poor self-reported health status at baseline (n=8,399) for the associations between sleep score and risk of health span termination** | | | | |
| --- | --- | --- | --- | --- |
| **Sleep score** | **Total N (%)** | **Cases N (%)** | **Basic model^a^** | **Fully adjusted model^b^** |
|  |  |  | **HR (95%CI)** | **HR (95%CI)** |
| 1 | 12896 (4.05) | 1999 (15.5) | ref | ref |
| 2 | 61125 (19.22) | 8238 (13.48) | 0.89 (0.85-0.93) | 0.93 (0.89-0.98) |
| 3 | 121752 (38.28) | 15102 (12.40) | 0.83 (0.79-0.86) | 0.91 (0.87-0.95) |
| 4 | 98402 (30.94) | 11159 (11.34) | 0.78 (0.75-0.82) | 0.89 (0.85-0.93) |
| 5 | 23881 (7.51) | 2480 (10.38) | 0.76 (0.72-0.80) | 0.89 (0.84-0.94) |
| continuous | 318056 (100.00) | 38978 (12.26) | 0.94 (0.93-0.95) | 0.97 (0.97-0.98) |

N, number; Hazard ratio, HR; 95%CI, 95% confidence interval; ref, reference;

**^a^:** Basic model, adjusted for age, sex and ethnicity;

**^b^:** Fully adjusted model, additionally adjusted for Townsend Deprivation Index, education, BMI, smoking status, alcohol consumption, physical activity, healthy diet, family history of diseases (cancer and CCVD) and medication (sleep-related drugs and aspirin/ ibuprofen)

| **Table S6. Sensitivity analyses for the associations between sleep score and risk of health span termination by further adjustment for PC1-3 and genotype chip (n=321,835)******* | | | | | |
| --- | --- | --- | --- | --- | --- |
| **Sleep score** | **Total (%)** | **Cases (%)** | **Basic model^a^** | **Fully adjusted model^b^** | **Further adjustment for  PC1-3 and genotype chip** |
|  |  |  | **HR (95%CI)** | **HR (95%CI)** | **HR (95%CI)** |
| 0-1 | 13127 (4.08) | 2482 (18.91) | ref | ref | ref |
| 2 | 62059 (19.28) | 10339 (16.66) | 0.85 (0.81-0.89) | 0.90 (0.86-0.94) | 0.90 (0.86-0.94) |
| 3 | 123255 (38.30) | 18896 (15.33) | 0.77 (0.74-0.81) | 0.87 (0.83-0.90) | 0.87 (0.83-0.90) |
| 4 | 99364 (30.87) | 13956 (14.05) | 0.72 (0.69-0.75) | 0.84 (0.81-0.88) | 0.85 (0.81-0.88) |
| 5 | 24030 (7.47 ) | 3085 (12.84) | 0.70 (0.66-0.73) | 0.84 (0.79-0.88) | 0.84 (0.80-0.89) |
| Continuous | 321835 (100.00) | 48758 (15.15) | 0.92 (0.91-0.93) | 0.96 (0.95-0.97) | 0.96 (0.96-0.97) |

**^*^:**7,015 participants with missing genetic data among 328,850 participants;
N, total number of observations; Hazard ratio, HR; 95%CI, 95% confidence interval; ref, reference; PC, principal component;

**^a^:** Basic model, adjusted for age, sex and ethnicity;

**^b^:** Fully adjusted model, additionally adjusted for Townsend Deprivation Index, education, BMI, smoking status, alcohol consumption, physical activity, healthy diet, family history of diseases (cancer and CCVD) and medication (sleep-related drugs and aspirin/ibuprofen).

**Supplementary Figure 1.** **Study design and workflow**

**Supplementary Figure 2. Stratification analysis for risk of health span termination by sleep score.** The median value of Townsend Deprivation Index is -2.28. Participants with sleep score 3-5 were compared to participants with sleep score 0-2 (reference) in stratification analyses. The ever smokers include previous and current smokers. Abbreviations: N, number; HR, Hazard ratio; 95%CI, 95% confidence interval; *P*-het, *P* value for heterogeneity.


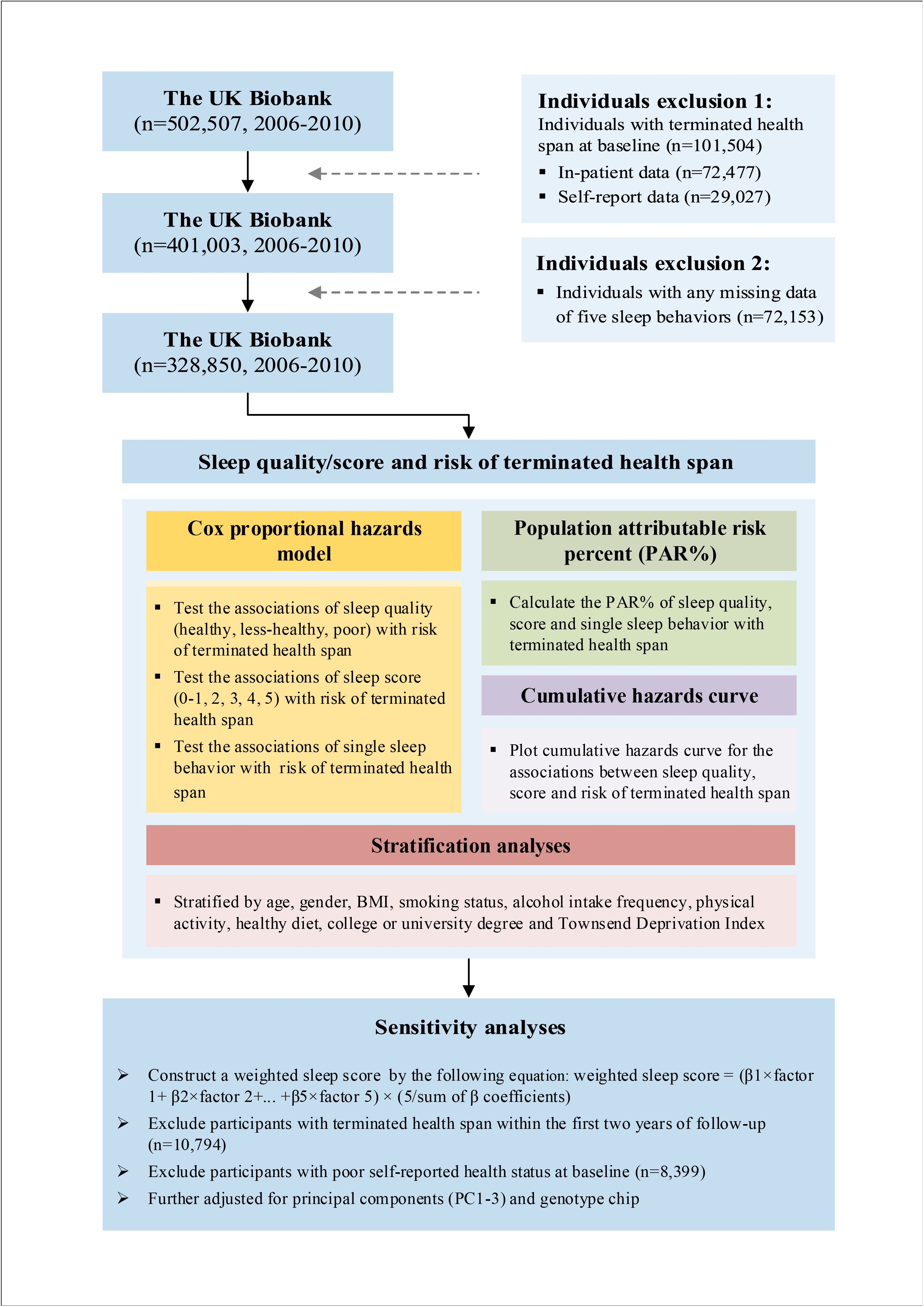
**Supplementary Figure 1.**

**Supplementary Figure 2.**

**
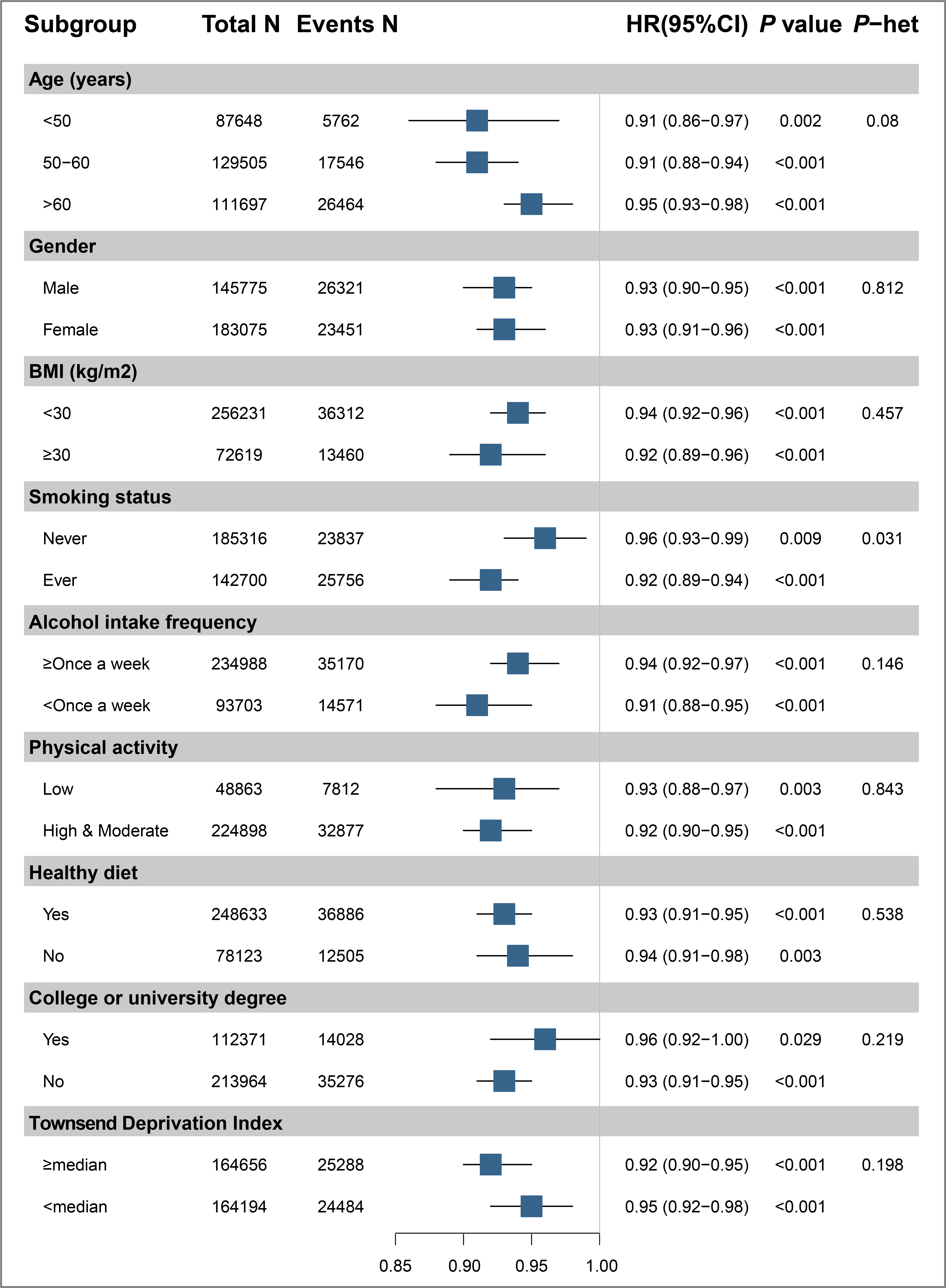
**
