# Supplementary figures and images for: Associations Between Sleep Quality and Health Span: A Prospective Cohort Study Based on 328,850 UK Biobank Participants
Source: Front Genet. 2021 Jun 15;12:663449. doi: 10.3389/fgene.2021.663449 (PMC8239359; doi:10.3389/fgene.2021.663449)

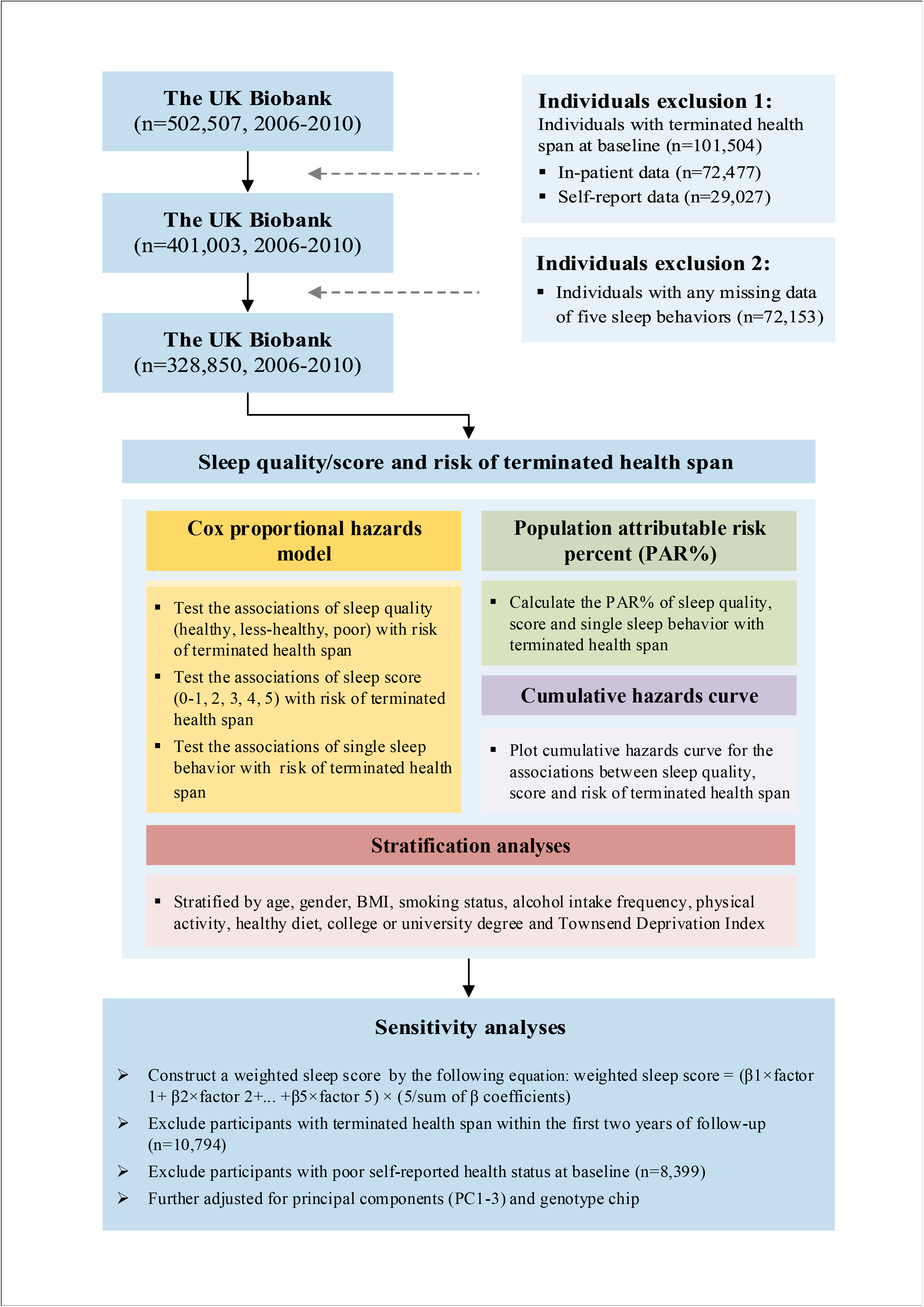

Supplement: Supplementary file 2 [file Image_1.tif]

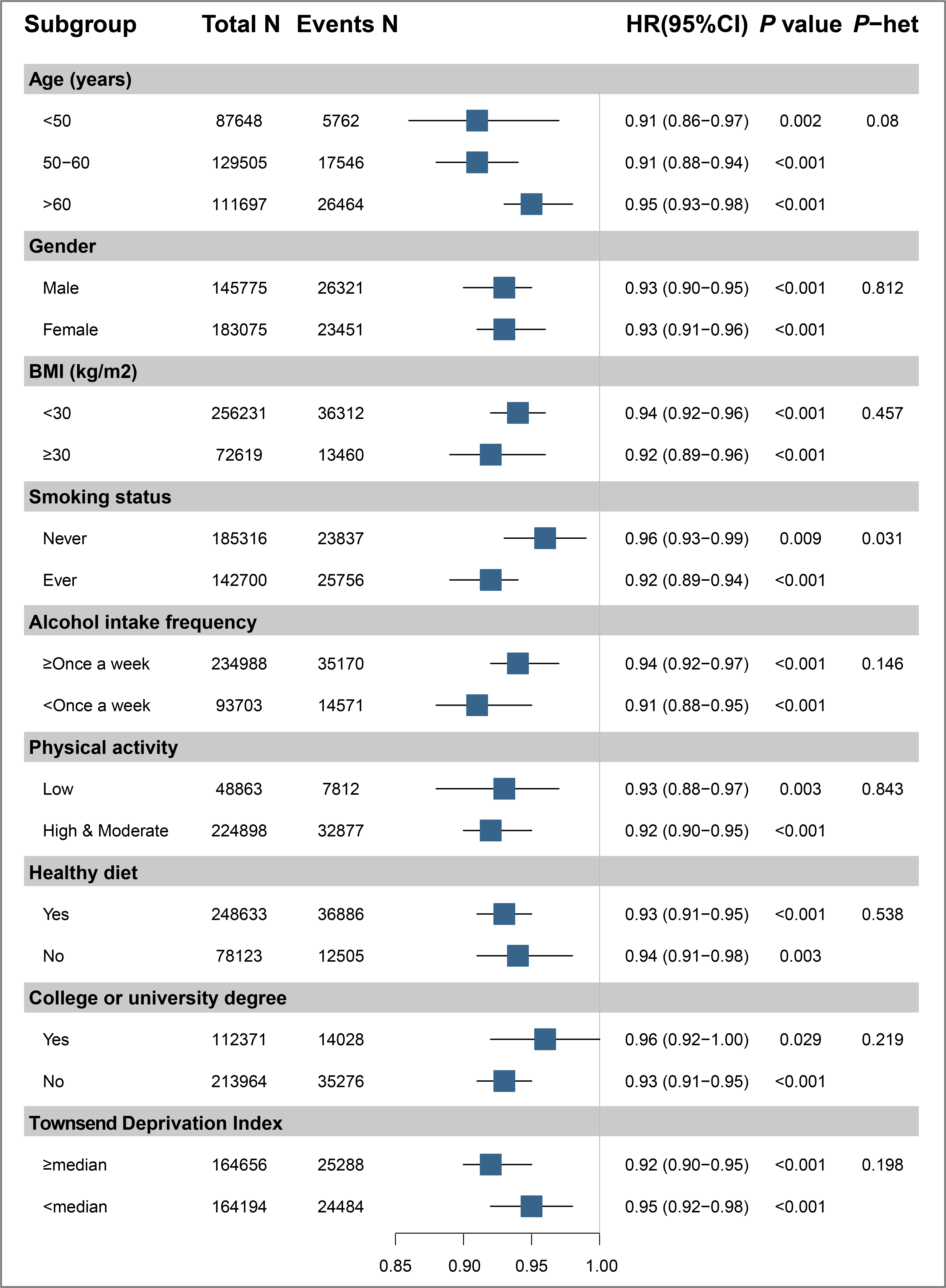

Supplement: Supplementary file 3 [file Image_2.tif]
